# Obituary for Joachim Robert Kalden

**DOI:** 10.31138/mjr.32.1.92

**Published:** 2021-03-30

**Authors:** Haralampos M. Moutsopoulos

**Affiliations:** Professor Emeritus and Member, Academy of Athens

## In memoriam

**Joachim Robert Kalden,** Born 23, 1937 in Marburg; †passed away February 6, 2021 in Erlangen.

The VIII European Workshop for Rheumatology research held in Corfu, Greece, in the spring of 1988, gave the opportunity to a handful of experts on Autoimmune Rheumatic disorders (ARDs), from different European counties, to constitute a group with a main goal to transform EULAR from a physical medicine and orthopaedics society to a contemporary League, supporting research towards understanding the pathogenesis and development of novel therapies for ARDs. This group consisted of Professors Ravinder Maini, JR Kalden, LBA van de Putte, Allan Wiik, I Lundberg, Lars Klareskog, J Smolen, P Youinou, and myself. I invited these experts for further discussions towards the materialisation of our goal, in a follow up meeting. For everyone’s convenience, this meeting was held close to the Athens airport, in the Astir palace hotel in Vouliagmeni, Attiki, Greece. Among these experts, a leading role was held by the now late Joachim Robert Kalden, a Lupus expert and subsequently a member of the leading group that introduced anti-TNF therapy for rheumatoid arthritis. Professor Kalden developed in Erlangen a world-renowned and respected centre for diagnosis, understanding the pathogenesis of lupus and application of novel therapeutic interventions in ARDs. In addition to research, Johan has edited, in collaboration with other colleagues, three books: (1) Progress in Immunology: Vol. VII: Proceedings of the 7th International Congress of Immunology Berlin 1989; (2) Rheumatoid Arthritis: Recent Research Advances in 1992; and (3) Der IL-1-Rezeptor-Antagonist im Zytokin-Netzwerk: Funktion und Stellenwert in 2013. Furthermore, he shared his knowledge and accomplishments with pleasure, everywhere he was invited to speak.

These memoirs came to my mind when I heard from Prof J Smolen that our beloved colleague and one of the founders of research centres in Europe for the study of ARDs, lost the war after a prolonged fight with SARS-COV2.

We will remember him as a well-organized personality, a thoughtful investigator, a didactic teacher, and sincere mentor for the development of younger colleagues as clinicians, therapists, and researchers.

We, his friends and colleagues, lost a great friend, an advisor in difficulties, and a joyful companion to have a glass of wine with good food. The Aegean Sea and islands will miss a lover of their unique bright light, blue sky and waters. Johan, we will always remember you.

**Figure:**
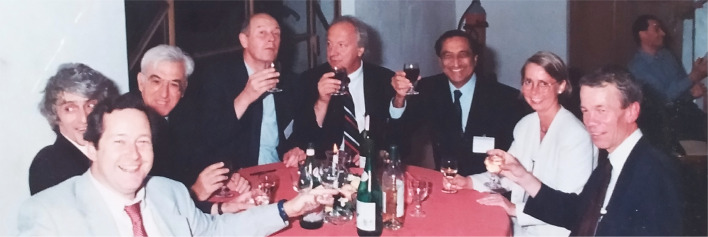
From left to right: Professors J. Smolen, P. Youinou, H. M. Moutsopoulos, L. Van De Patte, J. R. Kalden†, R. N. Maini, I. Lundberg, and L. Klareskog. (Astir Palace Vouliagmeni, Attiki, Greece, 1988)

